# The Rajarata Pregnancy Cohort (RaPCo): study protocol

**DOI:** 10.1186/s12884-020-03056-x

**Published:** 2020-06-26

**Authors:** Thilini Chanchala Agampodi, Nuwan Darshana Wickramasinghe, Rampathige Indika Ruwan Prasanna, Malawara Kankanamalage Lasandha Irangani, Jayasundara Mudiyanselage Samarakoon Banda, Pradana Mudiyanselage Bandula Jayathilake, Ayesh Hettiarachchi, Gayani Amarasinghe, Imasha Jayasinghe, Iresha Koralagedara, Sajaan Praveena Gunarathne, Sujanthi Wickramage, Janith Warnasekara, Niroshan Lokunarangoda, Vasana Mendis, Ajith Kumara Dissanayaka, Jagath Premadasa, Nandana Hettigama, Dayaratne Koralagedara, Manjula Weerasinghe, Krishanthi Malawanage, Hemali Jayakodi, Anuprabha Wickramasinghe, Suneth Buddhika Agampodi

**Affiliations:** 1 Department of Community Medicine, Faculty of Medicine and Allied Sciences, Rajarata University of Sri Lanka, Anuradhapura, Sri Lanka; 2 Department of Social Sciences, Faculty of Social Sciences and humanities, Rajarata University of Sri Lanka, Anuradhapura, Sri Lanka; 3 Department of Humanities, Faculty of Social Sciences and Humanities, Rajarata University of Sri Lanka, Anuradhapura, Sri Lanka; 4 Department of Environmental Management, Faculty of Social Sciences and Humanities, Rajarata University of Sri Lanka, Anuradhapura, Sri Lanka; 5 Department of Business management, Faculty of Management studies, Rajarata University of Sri Lanka, Anuradhapura, Sri Lanka; 6 Department of Anatomy, Faculty of Medicine and Allied Sciences, Rajarata University of Sri Lanka, Anuradhapura, Sri Lanka; 7 Department of Physiology, Faculty of Medicine and Allied Sciences, Rajarata University of Sri Lanka, Anuradhapura, Sri Lanka; 8 Department of Medicine, Faculty of Medicine and Allied Sciences, Rajarata University of Sri Lanka, Anuradhapura, Sri Lanka; 9 Department of Pathology, Faculty of Medicine and Allied Sciences, Rajarata University of Sri Lanka, Anuradhapura, Sri Lanka; 10 Department of Obstetrics and Gynaecology, Faculty of Medicine and Allied Sciences, Rajarata University of Sri Lanka, Anuradhapura, Sri Lanka; 11 Teaching Hospital Anuradhapura, Anuradhapura, Sri Lanka; 12 Regional director of Health Services Office, Anuradhapura, Sri Lanka; 13 Provincial Director of Health Services Office, Anuradhapura, Sri Lanka; 14 Department of Psychiatry, Faculty of Medicine and Allied Sciences, Rajarata University of Sri Lanka, Anuradhapura, Sri Lanka

**Keywords:** Pregnancy, Cohort, Sri Lanka, Social determinants, Mental health

## Abstract

**Background:**

Ending preventable maternal deaths remains a global priority and in the later stages of obstetric transition, identifying the social determinants of maternal health outcomes is essential to address stagnating maternal mortality rates. Countries would hardly achieve the Sustainable Development Goal (SGD) targets on maternal health, unless the complex and context-specific socio-economic aetiologies associated with maternal mental health and suicide are identified. The Rajarata Pregnancy Cohort (RaPCo) is a prospective cohort study, designed to explore the interactions between social determinants and maternal mental health in determining pregnancy and new-born outcomes.

**Methods:**

The study will recruit all eligible pregnant women in the maternal care programme of Anuradhapura district, Sri Lanka from July to September 2019. The estimated sample size is 2400. We will assess the socio-demographic and economic status, social capital, gender-based violence and mental health, including a clinical examination and biochemical investigations during the first trimester. Participants will undergo four follow-ups at 2nd and 3rd trimesters, at delivery and in early postpartum. The new-borns will be followed up at birth, neonatal period, at 6 six months and at 1 year. Pregnancy and child outcome data will be collected using direct contact. Qualitative studies will be carried out to understand the complex social factors and behavioural dimensions related to abortion, antenatal depression, maternal deaths and near misses.

**Discussion:**

This is the first reported maternal cohort in Sri Lanka focusing on social determinants and mental health. As a country in stage four of obstetric transition, these findings will provide generalizable evidence on achieving SGD targets in low- and middle-income countries. The study will be conducted in a district with multi-cultural, multi-ethnic and diverse community characteristics; thus, will enable the evidence generated to be applied in many different contexts. The study also possesses the strength of using direct participant contact, data collection, measurement, examination and biochemical testing to minimise errors in routinely collected data. The RaPCo study will be able to generate evidence to strengthen policies to further reduce maternal deaths in the local, regional and global contexts particularly focusing on social factors and mental health, which are not optimally addressed in the global agenda.

## Background

Every day, an average of 830 pregnant women around the globe die due to preventable causes [1]. Despite the drastic reduction in maternal mortality by 45% during the period of 1990–2015 [2], the global maternal health agenda is still facing major challenges and Ending Preventable Maternal Mortality (EPMM) is far from the reach [3]. The Millennium Development Goal (MDG) target of achieving annual rate of reduction (ARR) in maternal mortality ratio (MMR) of 5.5% was not a success. However, this target pushed the countries around the world to increase ARR from 1.1% in 1990–2000 period to 4.1% in 2000–2010 period [4]. Currently, the Sustainable Development Goal (SGD) 3 targets to reduce maternal mortality in the world to 70/100000 live births [3].

### Maternal health and social capital

With the “obstetric transition”, causes of maternal deaths are shifting from communicable diseases and direct obstetric causes such as sepsis, haemorrhage, pulmonary embolism (phase 1) to non-communicable diseases and indirect causes [5] (phase 4 and 5). High-income countries as well as some of the middle-income countries are in phase four and the shift is clearly demonstrated in the most recent maternal death estimates [6]. However, further reduction of maternal deaths has been a challenge and stagnation of MMRs is observed at global level [7].

Social development is identified as a prime to further reduce MMR in the latter phases of obstetric transition. Yet, the global maternal health agenda is primarily focused on medical interventions, neglecting the effects of social determinants and social interventions as root causes of all proximal determinants. Some aspects of social determinants, such as social capital in pregnancy and postpartum, are discussed only in few instances in global literature. “Social capital” in general reflects the state of social interactions among people. It is defined as the “features of social organization, such as trust, norms and networks that can improve the efficiency of society by facilitating coordinated actions” [8]. Social capital is a relatively new concept widely used in the field of social sciences [9, 10]. More recently, with the rise in global attention towards social determinants of health, social capital is considered as an important determinant that can influence the health of individuals and communities [11–14]. Few available studies on social capital and maternal health show that high social capital during pregnancy is associated with higher levels of self-rated health [11], lower levels of postpartum psychosis [15] and health related behaviours [16]. Social capital is also associated with health related behaviours in pregnancy [17]. It is identified that social capital in pregnancy is unique and it mainly encompasses cognitive and structural bonding in the immediate micro community of pregnant women. It is also hypothesized that these strong social ties exert a considerable impact on psychosocial wellbeing of pregnant women and having a significant inverse relationship to antenatal and postpartum anxiety and depression [17]. Micro-geographical variations of social capital is observed through ecological studies and may partly explain the health inequalities [18]. However, the predictive ability of social capital on pregnancy outcome has not yet been assessed.

### Maternal mental health and suicides

Globally, maternal suicide has emerged as a leading cause of maternal deaths over the past few decades [19]. It has become a leading cause of maternal deaths in some countries [20–22]. Suicide is only the tip of the iceberg. A recent systematic review shows that the suicidal ideation is largely underreported and the available tools are not capturing the actual risk [23]. Pregnancy and childbirth represent a time of increased vulnerability, during which a woman is exposed to many physiological and psychosocial changes, which put pregnant and postpartum women at increased risk of mental health problems. It has been consistently shown that the prevalence of maternal mental health issues are much greater in low-income countries (LIC) and lower-middle-income countries (LMIC) (16% and 20%, respectively) [24] than in high-income countries (HIC) [25]. The variability in estimates, seen especially in LIC and LMIC, is due to the differences in diagnostic criteria and timing of screening [26], socio-cultural and biological factors [27], and availability of data [25]. Of the mental health problems experienced by pregnant and postpartum women, antenatal depression, anxiety, and post-partum depression are the most common [28].

Antenatal depression, maternal anxiety and lack of psychosocial support during pregnancy is associated with low birth weight (LBW)/preterm birth (PTB), reduced gestational age at birth or intra uterine growth retardation (IUGR) [29–31]. Maternal depression has long-term effects on child development [32], poor mother-infant interaction [33], negative effects in the infant [34], and problems with cognitive development [35]. It is also shown that affective disorders [36] and attention-deficit/hyperactivity disorder (ADHD) are more common among children and adolescents of depressed mothers [37]. Multiple investigations have also found that women who experience antenatal depression are more likely to experience postpartum depression (PPD) as well [38–40].

### Sri Lankan scenario

In Sri Lanka, reanalysis of maternal deaths using International Classification of Diseases for Maternal Mortality (ICD MM) [41], the ICD version recommended for causes of maternal deaths classification, showed that suicide is the leading cause of maternal deaths in this area [42]. Further, previous research showed that 16.2% [27] of pregnant women are having depression and anxiety, and 27.1% of postpartum women are having depression [43]. In this given context, to achieve EPMM, suicide prevention and maternal mental health promotion should be considered as a high priority in Sri Lankan public health settings. Tackling this problem needs evidence on what determines maternal mental health at local level. Even though the global evidence base provides an overview of determinants of maternal suicide, it is crucial to consider the cultural and context-specific determinants in order to effectively promote maternal mental health. Further, with a view of capturing the dynamics of health-related behaviours, it is important to generate evidence based on longitudinal studies rather than solely based on cross-sectional studies. The more recent studies on exploring social capital and maternal health provided extensive data on the probable effects of social capital on maternal mental health. So far, there are no reported objective evaluations of social capital and maternal mental health conducted using prospective study designs. For evidence informed decision-making, high quality data based on robust methodologies are required. The best evidence for this type of outcome assessments comes though cohort studies. The Sri Lankan health system is one of the best in LMIC in maternal care and it usually captures more than 95% of pregnant women through public health pregnancy registration system [44]. This system provides a good opportunity to establish community cohorts. The aim of the proposed study, which is the Rajarata Pregnancy Cohort (RaPCo), is to evaluate the possible associations and interactions of maternal mental health and maternal social capital with the pregnancy outcome, neonatal outcomes and early childhood health and development outcomes in the Anuradhapura district, in order to aid in developing evidence-based guidelines on EPMM.

## Methods

### Aims

1. To determine the aetiological associations of mental health during pregnancy with pregnancy outcomes, neonatal outcomes and early childhood health and development outcomes.
2. To determine the possible interactions of complex social issues, social capital, socio-economic position, personality and gender-based violence on health-related issues during pregnancy, pregnancy outcomes, neonatal outcomes and early childhood outcomes.

### Study hypotheses (Fig. 1)

1. Social determinants such as poor social capital, poor socio-economic position and gender-based violence are independent risk factors of perinatal anxiety and depression.
2. Social capital of pregnant women is associated with anxiety and depression during pregnancy at both individual and ecological levels.
3. Mental health during early and late pregnancy is associated with pregnancy outcomes such as intra uterine growth retardation preterm labor and low birth weight.
4. Mental health during pregnancy and postpartum periods is a risk factor for non-exclusive breast feeding.
5. Mental health during pregnancy and postpartum periods is a risk factor for growth and development during infancy.
6. Social determinants such as poor social capital, poor socio-economic position and gender-based violence are independent risk factors of pregnancy and early childhood outcomes.
7. Abortion is associated with subsequent anxiety and depression.
8. Health system defaults such as unmet need and failure to follow-up for risk conditions are associated with adverse perinatal outcomes.
9. Intra-district micro-geographical variations exist in social determinants of maternal health and influence perinatal outcomes.
10. Differentials in social determinants has a causative impact on inequity of maternal and early childhood health and wellbeing.

**Fig. 1:**
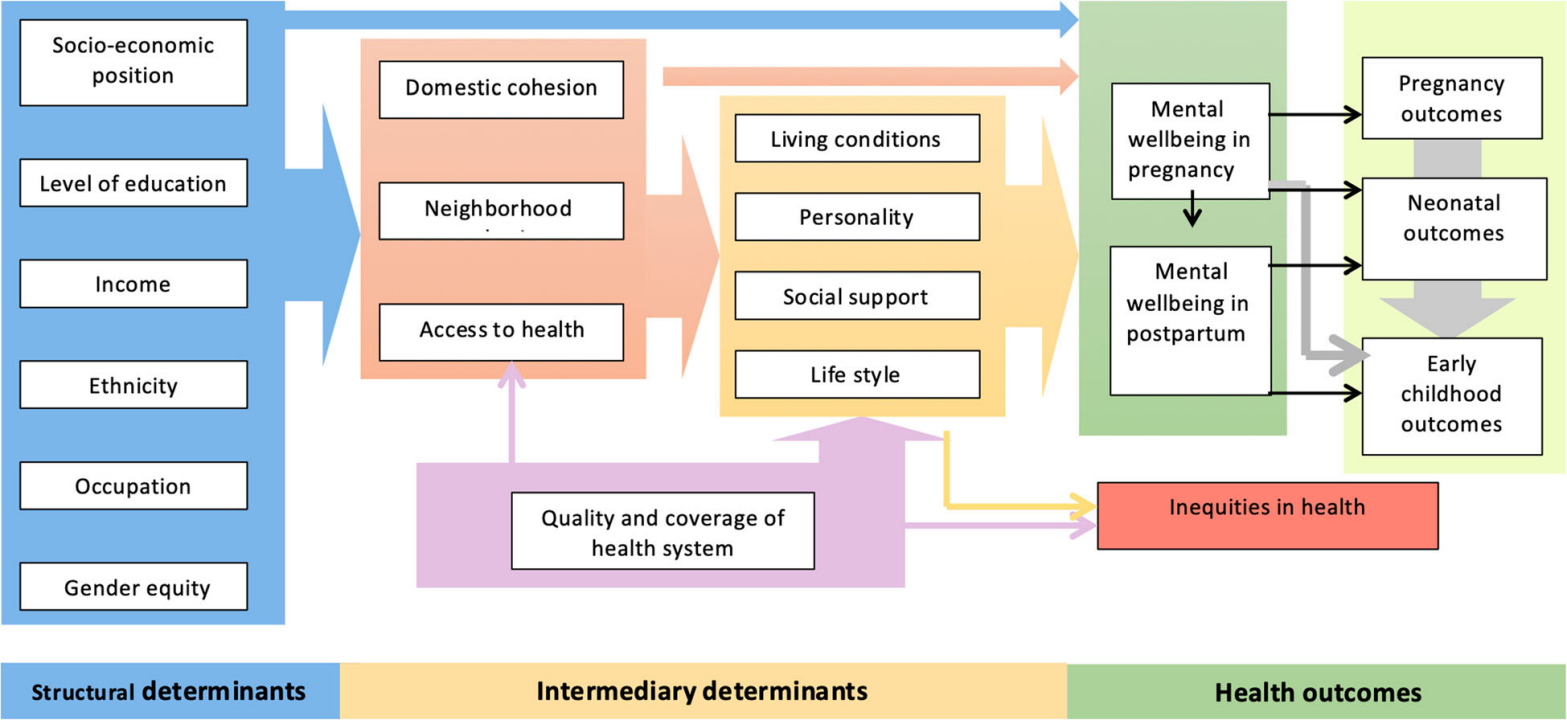
Conceptual framework of RaPCo study hypothesis

### Study design

This study is a prospective cohort study of pregnant women. Pregnant women will be recruited to the study in their early pregnancy (before 12 weeks) where they will undergo baseline assessment and will be followed up in the second and third trimesters in the field clinics and at delivery in the hospitals. Women will be followed up in the postpartum period in the field and infants will be followed up at routine child welfare clinics up to 1 year of age.

### Study setting

**Fig. 2:**
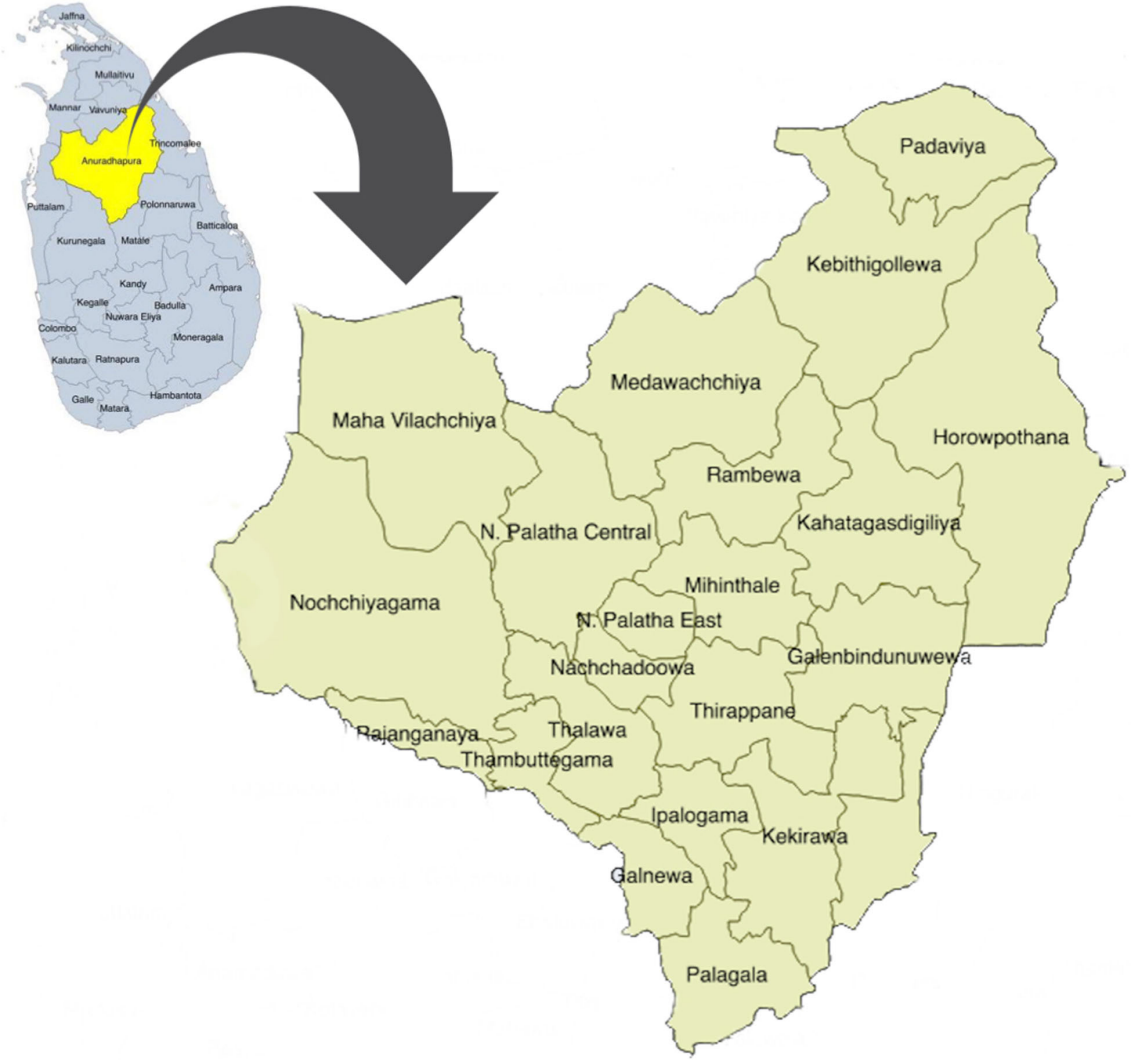
Spatial distribution of 22 participant recruitment areas of RaPCo study in the Anuradhapura district, Sri Lanka

The proposed study will be carried out in the Anuradhapura district (Fig. 2), which is the largest district in Sri Lanka. According to the routinely reported data, the resident population is 902930 with a birth rate of 17.8/1000 population [45]. The total fertility rate is 2.4 and the median age at first birth is 23.9 years, which is one of the lowest in Sri Lanka [46]. The number of pregnant mothers registered in the area in 2015 was more than 17000 and of them, 82.3% registered in field clinics before the eighth week of pregnancy and 96.0% had at least one clinic visit before delivery. The number of live births was 15376.

The services for pregnant women are provided through medical officer of health (MOH) and currently the district is divided in to 22 MOH areas. MOH is a community physician delivering public health services to the respective residing population. The grass-root level maternal and child health services are delivered through public health midwifes (PHM). Each MOH is consisted of 8–24 PHM areas. The PHMs are supervised by the Public Health Nursing Sisters (PHNS) (one PHNS per MOH each area). The district as a whole is divided in to 275 PHM areas with each PHM area is having a population of 1500–4000 people. PHMs register all pregnant women in the respective PHM areas in the routine maternal care service in the country in a register known as “Pregnant Mothers’ Register”. They provide domiciliary care at home visits and clinic care at antenatal clinics for all pregnant women. Curative services are provided through 56 hospitals/primary care units, including one teaching hospital (tertiary care) and three base hospitals (secondary care). Almost all deliveries (99.5%) are taken place in government hospitals [45].

### Study population

The study population for this study will include all pregnant women residing in the Anuradhapura district.

### Study sample

All pregnant women registered in pregnant mothers’ registers during the period of 1st July to 30th September 2019 in the maternal care programme in the Anuradhapura district will be recruited as the study participants.

#### Inclusion criteria

1. Pregnant women registered in pregnant mothers’ register of PHM and have completed the booking visit (first clinic visit) in antenatal clinics in the Anuradhapura district.
2. Permanent residence in the Anuradhapura district during the year ahead.
3. Period of amenorrhoea (POA)/gestational age (GA) less than 12 weeks by the time of recruitment.

#### Exclusion criteria

1. Pregnant women planning to leave the study area for childbirth or after the childbirth.

### Sample size

For the sample size calculation, we used a prevalence of antenatal depression of 16.2% as reported in Anuradhapura 2011 [27]. The relative risk [RR] of antenatal depression on for LBW, and IUGR reported in previous meta-analysis was 1.49 [95% CI = 1.25–1.77] and 1.45 [95% CI = 1.05–2.02] respectively [47]. We used the lowest RR for this calculation.

We used the sample size formula for Cohort studies as given by Kelsy et al. [48].

$${n}_1=\frac{{\left({Z}_{\alpha /2}+{Z}_{1\beta}\right)}^2\overline{p}\overline{q}\left(r-1\right)}{r{\left({p}_1-{p}_2\right)}^2}$$

where, n_1_ = number of exposed, n_2_ = number of unexposed, Z_α/2_ = standard normal deviate for two-tailed test, alpha level was taken as 90%, Z_β_ = standard normal deviate for one-tailed test based on beta level of 80%, r = ratio of unexposed to exposed 1: 0.16, p_1_ = proportion of pregnant women with depression having a bad pregnancy outcome (0.229), q_1_ = 1-p_1_, p_2_ = proportion of healthy pregnant women with bad pregnancy outcome (0.17), q_2_ = 1-p_2_, $\overline{p}=\frac{p_1+r{p}_2}{r+1}$, $\overline{q}=1-\overline{p}$. The calculated minimal sample size is 1948. With an anticipated loss to follow-up of 20%, the required size of the sample is computed to be 2338. We will recruit all pregnant women within a period of three months starting from July 2019 and the estimated sample will be around 2400.

### Recruitment of study participants

**Fig. 3:**
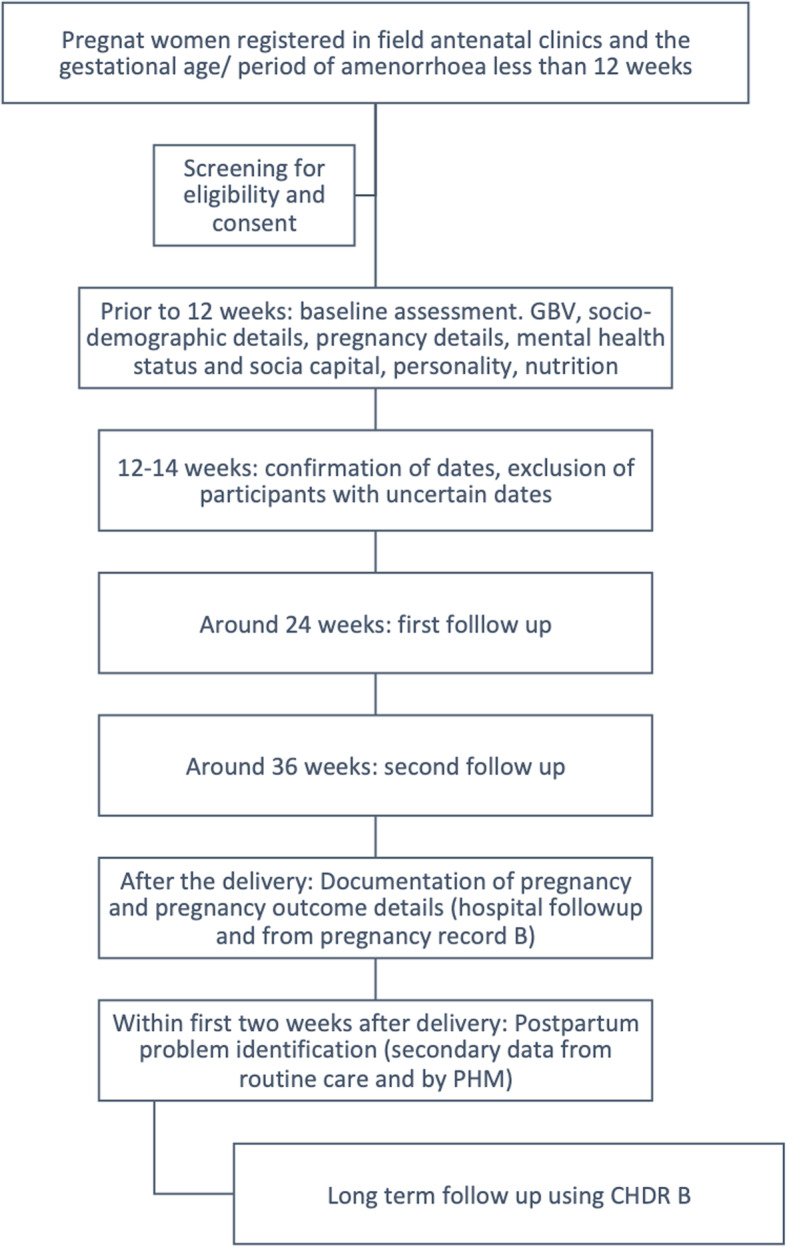
RaPCo study flow chart

RaPCo study flow chart (Fig. 3) and the participant timeline (Table 1) indicates the timing and procedures of the study from participant recruitment to close-out. The process of recruitment of participants will be mediated at the district level after explaining and informing the district level health authorities in order to initiate and maintain a strong baseline component. Advocacy meetings will be held to inform all 22 MOHs and the PHNS at district level and for the PHMs at divisional level using the routine monthly public health conference gatherings. Advocacy procedures will include strategic and systematic procedures including project presentations, video clips and unique information leaflets to all stakeholders including MOHs, PHNSs, PHMs and pregnant women. The field PHMs will do the initial introduction to the eligible pregnant mothers about the study. Once the pregnant women are registered and have completed the booking visit, they will be offered a date and the venue for the first contact with the study team using an invitation to a special clinic. The clinic schedules and sites will be predetermined at district and divisional level and on the consensus of all public health authorities. Pregnant women will be given a special set of instructions to bring all previous medical records and also to attend the clinic with fasting for 8 h to conduct necessary blood investigations. A team of researchers and healthcare providers from the public health system will jointly conduct the special clinic. A team will consist of an MBBS qualified medical officer, two pre-intern medical officers, four second-year medical undergraduates trained on data collection and a trained nurse for drawing blood samples. Eligible pregnant women will be given an explanation on the study objectives and informed written consent will be sought before the recruitment. Once consented, initial registration to the cohort will be done. All routine sample collection for anaemia, grouping and Rh, VDRL, urine albumin and sugar will be done during this clinic. OGTT test will be performed for all pregnant women, who are not already having a diagnosis of diabetes mellitus. While waiting for the blood sample collection of the 2nd hour test, data collection and health education will be carried out. Fully trained interviewers will collect data from the participants. Clinical examination will be done by MBBS qualified medical practitioners during this clinic session.

**Table 1:** Patient timeline of Rajarata Pregnancy Cohort

STUDY PERIOD								
	Enrolment	Allocation	Post allocation					
**Time point**	-t1 6–8 w	t0 12w	t1 25-28w	t2 32-36w	t3 Delivery	t4 Postpartum	t5 One year	Close-out
**Enrollment**	x							
**Eligibility Screen**	x							
**Informed consent**	x							
**Recruitment**		x						
Socio-demographic information, gender-based violence, personality		x						
Past obstetric history Past Medical history		x						
Obstetric details of current pregnancy, Medical complications of present pregnancy, Mental Health, Income and expenditure		x	x	x				
Social capital		x	x					
Food security				x				
Maternal height, waist to hip ratio		x						
Maternal weight, general examination, cardio vascular variables		x	x	x				
Liver function tests, Serum cholesterol, urine full report, blood picture, High performance liquid chromatography, serum ferritin		x						
Blood glucose (OGTT)		x	x					
Hemoglobin, red cell indices		x	x					
Serum Cortisol			x					
Mode of delivery, birth weight, maternal complications, neonatal complications					x			
Details of breastfeeding					x	x		
Postpartum depression, loneliness, neonatal details						x		
Growth and development indicators							x	
Close out								x

### Baseline assessment

Several questionnaires and laboratory investigations will be carried out (as outlined below) to assess the health status of pregnant mothers at baseline. Basic socio-demographic data, pregnancy details, medical and drug history will be obtained using an interviewer-administered questionnaire. Maternal mental health status, social capital, personality type, nutrition intake and economic costs of pregnancy will be assessed using self-administered questionnaires. Other self-administered questionnaires will be handed over to the pregnant women to complete at home. These questionnaires will be collected during the next clinic visit due in the second trimester. We will use a bar-coding system for all study instruments and all self-administered questionnaires will be without the personal identification details, hence the confidentiality will be maintained. Personal identification details will be collected during the first interview and subsequently, only bar-coded tools will be used.

#### Study tools

We will use several study tools to assess the intended measures as described below.

1. Socio-demographic factors, medical history and pregnancy details: A study-specific interviewer-administered questionnaire prepared by the investigators in Sinhala and Tamil
2. Anxiety and Depression in pregnancy and postpartum: Validated Sinhala and Tamil versions of the Edinburgh Post-Partum Depression Scale (EPDS) [49]
3. Gender-based violence: Validated Sinhala and Tamil versions of Abuse Assessment Screen (AAS) [50]
4. Productivity cost due to maternal morbidities: Culturally adapted and validated Sinhala and Tamil versions of the WHO Impact Tool Kit (selected components) [51]
5. Personality: Culturally adapted and validated Sinhala and Tamil versions of Ten Item Personality Inventory (TIPI) [52]
6. Social capital in pregnancy and postpartum: Low and middle income countries Social Capital Assessment Scale related to maternal Health (LSCAT-MH) (originally developed and validated Sinhala and Tamil versions) [53]
7. Dietary assessment: Food frequency questionnaire and assessment of diet plate prepared by the investigators
8. Psychological distress: Validated Sinhala and Tamil of General Health questionnaire 12 [54]
9. Experience based house hold food security: Translated and adapted Sinhala and Tamil versions of Latin American and Carribbean Household Food Security Scale (ELCSA) [55] (This questionnaire was translated according to Sumathipala and Murrays technique [56] and an expert validation was performed using a panel of experts in public health and nutrition (see additional files one and two).

Since the literacy rate is 94.8% in the study area [57], we expect that the pregnant mothers will be able to complete the self-administered questionnaires. However, if there are any mothers having difficulties in completing the questionnaires on their own, data collectors will assist them in the procedure. Data extraction at follow-up visits will be done using data extraction forms prepared by the investigators.

#### Measurements and clinical examination

Anthropometric measurements will be collected using the standard protocols [58]. We will measure weight, height, waist and hip circumference during the clinic visit. Blood pressure measurement and the to pulse rate will be recorded using automated BP monitors. A general examination and auscultation of heart will be done using clinical examination guidelines and specific cardiac stethoscopes.

#### Biochemical tests

All routine biochemical analysis conducted at antenatal clinics will be offered through the RaPCo, except VDRL and grouping and Rh, which will be done in the reference hospitals in the Sri Lankan maternal health programme. The venepuncture will be done by an experienced, registered nurse using aseptic techniques and standard precautions, as a part of routine booking visit sample collection. Baseline biochemical parameters will include full blood count, plasma glucose, liver function tests, serum cholesterol and urine full report. A sample of whole blood, serum and urine will be collected. Samples will be immediately sent to the Public Health Research Laboratory of Faculty of Medicine and Allied Sciences, Saliyapura. Full blood count and plasma glucose levels will be done on the same day. A sample of urine will also be collected to a sterile bottle. Urine analysis will be done using 10 parameter strip test. An aliquot of whole blood, serum and processed urine will be stored at -80^0^c for batch-wise analysis.

### Follow-up visits

#### First follow-up visit (second trimester of pregnancy)

All pregnant women will be followed up in routine antenatal clinics/antenatal sessions of the Sri Lankan maternal care programme. Those who are not attending the clinics or antenatal sessions will be followed up at home. The first RaPCo follow-up visit will be done around 24 weeks of gestation (during the second trimester). All data pertaining to the pregnancy after the first contact will be obtained from the pregnancy record maintained in the maternal care system (H519 A portion) and an interviewer-administered questionnaire. In the second trimester, we will reassess pregnancy weight gain, blood glucose, haemoglobin level, social capital, mental health and health expenditure. Serum cortisol levels will be assessed in a sub-sample of women.

#### Second follow-up visit (third trimester of pregnancy)

Second follow-up visit will be similar to the first follow-up visit. This will be done around 32–36 weeks of gestation. Maternal mental health, food security, health expenditure and blood biochemistry related to the project objectives will be assessed during this visit.

#### Third follow-up (hospital follow up at delivery)

All pregnant women are given a special set of instructions to inform the research team regarding all hospital admissions during pregnancy. All admission related data will be obtained through a surveillance system set up for this purpose. Details of hospital admissions will be obtained from patient records and also through an interview during a special visit. In the third trimester, we will set up full time research assistants in two major hospitals (in which 90% of all deliveries in the area) to collect all hospital data and a small mobile team of researchers to collect data from small hospitals. A cord blood sample will be obtained from a sub-sample to assess the cord blood biochemistry as an intermediate variable for predictor and neonatal/infant outcome variables for a long-term follow-up. Data on the mode of delivery, maternal complications, birth weight, neonatal complications and establishment of breastfeeding will be collected by trained pre-intern medical officers.

#### Fourth follow- up visit (postpartum)

The pregnancy outcome and the neonatal outcome details will be complemented from the pregnancy record B portion and Child Health Development Record (CHDR) B portion, which are available with PHMs. Post partum depression will be assessed using EPDS 2 weeks after the delivery. This procedure is a part of routine care programme and will be done by PHMs. Social capital will be assessed using LSCAT- MH. Details on breastfeeding and neonatal complications will be further collected through PHMs.

#### Fifth follow-up (infants at 1 year)

The follow-up data after 1 year will be collected using the CHDR B portion available with the PHMs. The main focus would be the growth and development data.

#### Laboratory procedures

Sample analysis will be done in the Public Health Research Laboratory of the Department of Community Medicine, Faculty of Medicine and Allied Sciences, Rajarata University of Sri Lanka. All analysis will be done using a fully automated biochemistry analyser, according to the manufacturers guidelines. A fully qualified medical laboratory technician will be recruited for this purpose and internal and external quality assurance will be done as recommended for all tests.

### Qualitative exploration of social determinants of maternal health

In-depth qualitative studies will be conducted using relevant subsamples of participants to understand the real life situations and the dimensions of health behaviours related to abortion, antenatal depression and anxiety, maternal deaths and near misses. Identification of participants for the studies will be based on the baseline and follow up data collected in the cohort. In-depth interviews will be conducted according to standard protocols by trained interviewers and qualitative and social science experts in the team. We will be using narrative medicine approaches to understand the meaning, perspective and patient predicament to supplement the quantitative data on building an explanatory model to mental health issues in pregnancy related to health as well as social issues. Thematic analysis, mapping, narrations and explanatory model building will be used to identify the intended social mechanisms.

### Data management and analysis

The data will be available only for investigators and will be stored in password-protected computers accessible only to the investigators. A cloud-based, real-time data entry programme will be used for field data collection. The data entry will be optimized with field checks, data type restrictions and logical arguments imbedded in data entry programme. This database will be linked to the laboratory data through bar coding system.

Descriptive statistics will be computed using proportions and percentages to describe the baseline data. Loss to follow-up will be analysed separately to identify and describe the characteristics of these participants. EPDS analysis will be done as described in the EPDS guidebook and the threshold value of 9 will be used in this study to identify maternal depression and anxiety. Social capital score will be generated for different constructs and the constructs will be considered as variables. Incidence of abortions, still births, LBW, preterm deliveries, failure in exclusive breast feeding, growth faltering, common childhood illnesses and postpartum psychosis/depression will be calculated. The relative risk of poor social capital and mental health in pregnancy on the selected outcomes will be calculated with 95% confidence limits. Development of the tool to identify those who are with high risk for perinatal depression will be done using significant predictors identified at binary logistic regression. We will develop a conceptual framework based on baseline data before fitting the model.

### Data quality assurance

Data quality will be assured at field data collection by using standard protocols, training of data collectors, supervision and feedback. In databases planned random and frequency checking with systematic feedback will ensure the data quality during the study process. For qualitative components triangulation, standard techniques, respondent validation and reflexivity will be adopted to maintain trustworthiness of data.

### Ethical considerations

Being a cohort study, there will be a few ethical concerns in this study. Written informed consent will be taken from pregnant mothers to participate in this study as well as to use routinely collected data for this research purpose. The participants will be informed that this research is conducted in parallel with the routine maternal and child health service and any abnormal finding will be reported to the health provider with the participant’s consent. In addition, consent will be sought for the use of serum sample for screening of infections, serum cortisol level and any other future studies, that may require baseline assessment of serum. Data will be linked to the health records using a classified code, so that the confidentiality of data will be secured. Individual level data will not be presented in any form and summary data will be used in research publications and data dissemination.

The poor social capital identified through the questionnaire will need attention. As this is part of the routine duty of PHMs, we will inform the details to the PHM to follow-up these mothers as “risk” mothers (as per the guidelines in the routine maternal care programme in Sri Lanka) in their service delivery. Same procedure will be done for pregnant mothers with high EPDS score. These women will be referred to the MOH for a clinical interview and via the MOH, we will refer the mothers for appropriate psychiatric and/or counselling services. Mothers who are reported as having gender-based violence will be given special attention and counselling. At present, the public health system has a “red book”, which requires entering of these data and appropriate referral.

### Patient and public involvement and engagement (PPIE)

The development of the protocol for RaPCo was based on a lengthy PPIE approach. Over a period of more than 2 years we have been having formal and informal discussions with pregnant women, public health midwives, medical officers of health and all health care providers to prioritize the health needs of pregnant women in Anuradhapura. We consulted pregnant women through a formal process to include social, neighbourhood and community level socio-economic and cultural determinants to this study. Proposal development was based on another PhD study, which took place over several years, and the one of the main component was to identify research priorities related to maternal health through an eye of pregnant women. We collaborate with pregnant women at group meetings to express their views on research questions. A pregnant colleague is included as a co-investigator to look at the protocol critically. For the ethics application, pregnant women from the Anuradhapura formally reviewed patient information sheets/ consent forms and lay summary. The booklet prepared for pregnant women, which included some of the study tools were reviewed by pregnant women representing different social status and final product was edited based on the critical reviews. Beyond pregnant women, we had formal discussions with public health staff, three rounds of discussions with public health midwives, a formal meeting with medical officers of health, several meetings with medical officer maternal and child health (MOMCH), a meeting with secretory of health in North Central Province and also a meeting with consultant community physician (CCP) of the NCP. Informal discussions were held with two obstetricians working in the district and once the ethical clearance is obtained, we will have a full briefing meeting and a discussion with all consultant obstetricians to inform about the procedure.

During the research process, we will include pregnant women from different stages of gestation to get their perspectives, priorities and issues related to research problem and process. This will be done ensuring the privacy and confidentiality of participant information, at the same time empowering participants to be involved in the research process. We will include pregnant and postpartum women in preparation of public awareness programmes and public communication. As described in the methods, PPIE is embedded in this study through a social participatory research approach.

### Administrative concerns

The study is planned in collaboration with the Regional Director of Health Services (RDHS) Anuradhapura with the approval of Provincial Director of Health Services. The provincial consultant community physician and the MOMCH are collaborators of this study. As a part of this study, we will have workshops to improve record keeping, quality of data and data analysis workshops for all public health field staff.

## Discussion

In June 2019, pretesting of instruments and pilot testing of the project was conducted. Major changes were done after the pre-test and pilot test to improve the understanding of questions and to improve the quality of data and sample collection procedures. Several rounds of discussions were held with stakeholders to make sure that the project is well understood and supported by all stakeholders. Training of public health staff was also carried out to improve the data quality in routine documentation process. This seemed necessary after the preliminary work on data quality in pregnancy records. Several data fields were included to the questionnaires to support the development and assessment of public healthcare delivery system in the district. We have already completed the recruitment process from July to September 2019.

Provided that data from a large sample will be collected in a cohort of pregnant mothers, the proposed project has branched out to explore maternal health conditions over a period of time. For instance, at the moment, separate studies have been already planned on assessing heart disease, anaemia, metabolic syndrome, fatty liver and out of pocket expenditure in sub-sample of pregnant mothers included in the study. Given the wealth of information planning to be obtained in this cohort, we believe this proposed study would lead to numerous related studies in the future.

Further, longitudinal data generated from this proposed study would aid in better understanding of the complex dynamics of health-related behaviours among pregnant mothers and their effects, interrelationships with health related outcomes in the pregnant women and new-borns. According to the reported literature, our proposed study is the largest community-based maternal and child health cohort study in Sri Lanka, which have used an extensive PPIE in the development phase of the study. Hence, the study findings would not only be more accurate, but also would yield information, which would be of greater practical significance for the service delivery to the concerned community.

## Supplementary information

**Additional file 1.** Latin American and Carribbean Household Food Security Scale (ELCSA)- Translated and adapted Sinhala version. Translated and adapted Sinhala language tool

**Additional file 2.** Latin American and Carribbean Household Food Security Scale (ELCSA)- Translated and adapted Tamil version. Translated and adapted Tamil language tool

## Data Availability

The datasets generated during the current study will be available with the ERC, Faculty of Medicine and Allied Sciences, Rajarata University of Sri Lanka. Once the baseline data collection is completed, we will deposit data without personal identification in OSF platform (https://osf.io). Full data related to individual papers will also be published with papers as appropriate.

## Abbreviations

AAS: Abuse Assessment Screen
ADHD: Attention-Deficit/Hyperactivity Disorder
BP: Blood Pressure
CHDR: Child Health Development Record
CI: Confidence interval
CWC: Child Welfare Clinics
EPDS: Edinburgh Post-Partum Depression Scale
EPMM: Ending Preventable Maternal Mortality
GA: Gestational Age
GBV: Gender Based Violence
ICD-MM: International Classification of Diseases for Maternal Mortality
LBW: Low Birth Weight
LMIC: Low and Middle Income Countries
LSCAT_MH: Low and middle income countries Social Capital Assessment Scale related to maternal Health
MBBS: Bachelor in Medicine, Bachelor in Surgery
MMR: Maternal Mortality Ratio
MOH: Medical Officer of Health
MOMCH: Medical Officer, Maternal and Child Health
OGTT: Oral Glucose Tolerance Test
PHM: Public Health Midwife
PHNS: Public Health Nursing Sister
POA: Period of Amenorrhea
PPIE: Patient and Public Involvement and Engagement
RaPCo: Rajarata Pregnancy Cohort
RDHS: Regional Director of Health Services
SDG: Sustainable Development Goal
TIPI: Ten Item Personality Inventory
VDRL: Venereal Disease Research Laboratory
