# Supplementary material for: The Rajarata Pregnancy Cohort (RaPCo): study protocol
Source: BMC Pregnancy Childbirth. 2020 Jun 26;20:374. doi: 10.1186/s12884-020-03056-x (PMC7318435; doi:10.1186/s12884-020-03056-x)
Supplement: Supplementary file 1 — Additional file 1. Latin American and Carribbean Household Food Security Scale (ELCSA)- Translated and adapted Sinhala version. Translated and adapted Sinhala language tool [file 12884_2020_3056_MOESM1_ESM.pdf]

RaPCo අංකය

ගෙදර දොර ආහාර සුරක්ෂිතතාවය පිලිබඳ සොයා බැලීම

අපේ මේ සුදානම අම්මගේ ගෙදර ආහාර සුරක්ෂිතතාවය පිලිබඳ සොයල බලන්න. සරලව කියනවනම් මෙයින් අදහස් කරන්නේ ගෙදර සියලු දෙනාට ගුණදායක ආහාර අවශ්‍ය පමණ ගෙදර දොරේ තියනවද කියල බලන එක. **ආහාර සුරක්ෂිත්තාවය නොමැති වීමට හේතුන් ලෙස අත මිට මුදල් හිඟ වීම , තමන්ට අවශ්‍ය ප්‍රමාණයට අහාර නිපදවා ගැනීමට නොහැකි වීම, මිලදී ගැනීම සඳහා ළඟ පාත කඩවල සුදුසු ආහාර නොමැති වීම, සමාජ සම්බන්ධතා නැතිවීයාම, සහනාධාර නොමැතිවීම, නියඟය හෝ ජල සැපයුම නොමැති වීම හෝ දේශපාලන අස්ථාවර භාවය** ආදිය දක්වන්න පුළුවන්.

මෙහි ගෙදර අය යනුවෙන් අදහස් කරන්නේ කුඩා දරුවන් නොව අවුරුදු 18 ට වැඩි අය බව සලකන්න. <u>ගෙදර යම් කෙනෙක් කෑම තිබිලත් රුචිය නොමැති වීම නිසා හෝ තමන්ගේ බර අඩු කරගැනීමට කෑම පාලනය කරන නිසා කෑම නොගෙන සිටීම මෙහිදී **අදාල නොවන** බව ද කරුණාවෙන් සලකන්න.</u>

(මේ ප්‍රශ්නාවලියට ඔබ ලබාදෙන පිළිතුරු මත පදනම්ව ඔබට ආධාර හෝ වෙනත් වරප්‍රසාද ලබාදීම හෝ ඔබ දැන් ලබන වරප්‍රසාද අඩුවීම සිදුනොවන නිසාත් මෙය අනාගතයේ අපේ මවුවරුන්ගේ සහ දරුවන්ගේ යහපත සඳහා යොදාගත හැකි නිසාත් සත්‍ය තොරතුරු පමණක් අපට ලබා දීමට කාරුණික වන්න)

පසුගිය මාස තුනක කාලය තුල ගෙදර කෑම බීම තත්ත්වය ගැන අහල තියෙන පහත ප්‍රකාශ වලට අම්මගේ නිවසට අදාලව දිය හැකි උත්තරය රවුම් කොට දක්වන්න.

1. පසුගිය මාස 03 තුළ ගෙදර කෑම බීම හිඟ වෙයැයි ඔබ බියෙන් සැකෙන් සිටි අවස්ථා තිබුණාද ?	ඔව්	නැහැ	නොදනී
2. පසුගිය මාස 03 තුළ ගෙදර කෑම බීම හිඟ ව ගිය අවස්ථා තිබුණාද ?	ඔව්	නැහැ	නොදනී
3. පසුගිය මාස 03 තුළ ඉහත සඳහන් කල යම් හේතුවක් නිසා ඔබට හෝ ගෙදර අයට පෝෂණීය සහ ගුණදායක අහාර ලබාගැනීමට නොහැකිවූවාද ?	ඔව්	නැහැ	නොදනී
4. පසුගිය මාස 03 තුළ ඉහත සඳහන් කල යම් හේතුවක් නිසා ඔබට හෝ ගෙදර අයට විවිධත්වයක් නැතිව එකම කෑම වර්ග දිගින් දිගටම කෑමට සිදුවූ අවස්ථා තිබුණාද ?	ඔව්	නැහැ	නොදනී
5. පසුගිය මාස 03 තුළ කෑම මදි වෙයැයි බියෙන් ඔබට හෝ ගෙදර අයට උදේ, දවල් හෝ රෑ කෑම වේල් මගහැර සිටීමට සිදුවූ අවස්ථා තිබුණාද ?	ඔව්	නැහැ	නොදනී
6. පසුගිය මාස 03 තුළ කෑම හිඟව යාම නිසා ඔබ හෝ ගෙදර අය කෑමට අවශ්‍ය වී තිබුණ ප්‍රමාණයට වඩා අඩුවෙන් කා සෑහීමට පත්වූ වේල් තිබුණාද ?	ඔව්	නැහැ	නොදනී
7. පසුගිය මාස 03 තුළ ගෙදර කෑම බීම නොමැති වීම නිසා ඔබ හෝ ගෙදර අය බඩගින්න දැනී දැනී වේලක් නොකා අවස්ථා සිටි තිබුණාද ?	ඔව්	නැහැ	නොදනී
8. පසුගිය මාස 03 තුළ ගෙදර කෑම බීම නොමැති වීම නිසා ඔබ හෝ ගෙදර අය බඩගින්න දැනී දැනී මුළු දවසම වාගේ නොකා සිටි අවස්ථා තිබුණාද ?	ඔව්	නැහැ	නොදනී
