# Supplementary material for: The Rajarata Pregnancy Cohort (RaPCo): study protocol
Source: BMC Pregnancy Childbirth. 2020 Jun 26;20:374. doi: 10.1186/s12884-020-03056-x (PMC7318435; doi:10.1186/s12884-020-03056-x)
Supplement: Supplementary file 2 — Additional file 2. Latin American and Carribbean Household Food Security Scale (ELCSA)- Translated and adapted Tamil version. Translated and adapted Tamil language tool [file 12884_2020_3056_MOESM2_ESM.pdf]

RaPCo இலக்கம்

வீடு உணவு சுகாதாரம் குறித்த தேடுதல் விபரம்

எங்களது இந்த முயற்சியானது அம்மாவின் வீட்டு உணவு சுகாதாரத்தை பற்றிய விபரங்களை அறிவதாகும். இலகுவாக கூறுவதானால் இம்முயற்சியின் நோக்கமானது வீட்டிலுள்ள அனைவருக்கும் சுகாதாரமான உணவு போதிய அளவில் இருக்கிறதா என்பதை அறிவதற்கே ஆகும். உணவு சுகாதாரம் இல்லாமைக்கு காரணமாக பண தட்டுப்பாடு, தமக்கு போதிய அளவு உணவை சமைக்க முடியாமை, நல்ல உணவு வகைகளை வாங்குவதற்கு அருகாமையில் கடைகள் இல்லாமை, சமூக ஈடுபாடு இன்மை, மானியங்கள் கிடைக்காமை, வறட்சி அல்லது நீர் வசதி இல்லாமை அல்லது அரசியல் உறுதிபாடுகள் என்பவற்றை கூறலாம்

இங்கு வீட்டு உறுப்பினர்கள் என கூறுவது சிறுவர்களை அல்ல. 18 வயதுக்கு மேற்பட்ட நபர்களையே கூறுகிறோம். வீட்டில் ஒருவர் உணவு இருந்தும் பசியின்மை காரணமாகவோ தங்கள் எடையை குறைத்து கொள்வதற்காகவோ உணவு கட்டுபாட்டை கடைபிடிப்பாராணால் இவை இக்கணக்கில் எடுத்து கொள்ளப்பட மாட்டாது என்பதை கருத்திற்கொள்ளவும்.

(இந்த கேள்விகளுக்கு நீங்கள் தரும் பதிலின் அடிப்படையில் உணவு அல்லது வேறு வரப்பிரசாதங்கள் மற்றும் உங்களுக்கு தற்போது கிடைக்கும் வரப்பிரசாதங்களில் மாற்றங்கள் எதுவும் நடைபெறாது என்பதை தெரிவித்துக்கொள்கிள்றோம். இது நமது எதிர்கால தாய்மார்களின் மற்றும் குழந்தைகளின் நன்மைக்கு பயன்படுத்திக் கொள்ளப்படும்: ஆதலால் உண்மையான பதிலை தர முயலவும்)

கடந்த மூன்று மாத காலத்திற்குள் வீட்டில் உணவு பற்றி கீழே தரப்பட்டுள்ள வினாக்களுக்கு வீட்டை கருத்திற் கொண்டு கூற கூடிய சரியான பதிலை வட்டமிட்டு காட்டுக.

1. கடந்த மூன்று மாதத்தில் வீட்டிலுள்ள உணவு பண்டங்கள் பற்றாமல் போகும் என அச்சத்தில் இருந்ததுண்டா?	ஆம்	இல்லை	தெரியாது
2. கடந்த மூன்று மாதத்தில் உணவு பண்டங்கள் பற்றாமல் போன சந்தர்ப்பம் இருந்தது உண்டா?	ஆம்	இல்லை	தெரியாது
3. கடந்த மூன்று மாதத்தில் மேலே குறிப்பிட்டவாறு ஏதேனும் ஓர் காரணத்தினால் உங்களுக்கு அல்லது வீட்டிலுள்ள எவருக்காவது சத்துள்ள உணவை பெற முடியாமல் போனதுண்டா?	ஆம்	இல்லை	தெரியாது
4. கடந்த மூன்று மாதத்தில் மேலே குறிப்பிட்டவாறு ஏதேனும் ஓர் சந்தர்ப்பத்தில் உங்களுக்கு அல்லது வீட்டிலுள்ள எவருக்காவது வேறுபாடு இன்றி ஒரே உணவை அடிக்கடி உண்ண நேர்ந்ததா?	ஆம்	இல்லை	தெரியாது
5. கடந்த மூன்று மாதத்தில் உணவு பற்றாமல் போகுமோ என்ற அச்சத்தில் எப்போதாவது உணவு வேளையை தவிர்த்தீர்களா?	ஆம்	இல்லை	தெரியாது
6. கடந்த மூன்று மாதத்தில் உணவு பற்றாமல் போனதால் நீங்கள் அல்லது வீட்டிலுள்ளவர்கள் தேவையான அளவு உணவை விட குறைந்த அளவு உண்ணும் சந்தர்ப்பம் இருந்ததா?	ஆம்	இல்லை	தெரியாது
7. கடந்த மூன்று மாதத்தில் உணவு பற்றாமல் போகுமோ என்ற அச்சத்தில் பசி என அறிந்தும் உண்ணாமல் இருந்ததுண்டா?	ஆம்	இல்லை	தெரியாது
8. கடந்த மூன்று மாதத்தில் உணவு பற்றாமல் போவதால் நீங்கள் அல்லது வீட்டிலுள்ளவர் பசி ஏற்பட்டும் முழு நாளும் உண்ணாமல் இருந்தது உண்டா?	ஆம்	இல்லை	தெரியாது
